# Preoperative hip abductor strength predicts discharge destination after total hip arthroplasty

**DOI:** 10.1007/s00590-024-04119-x

**Published:** 2024-11-15

**Authors:** Shusuke Nojiri, Azusa Kayamoto, Chiaki Terai, Shinya Tanaka, Yusuke Osawa, Yasuhiko Takegami

**Affiliations:** 1 Department of Rehabilitation, Nagoya University Hospital, 65 Tsuruma-Cho, Showa-Ku, Nagoya, Aichi, 466-8550 Japan; 2 Department of Orthopaedic Surgery, Nagoya University Graduate School of Medicine, Nagoya, Japan

**Keywords:** Total hip arthroplasty, Discharge destination, Preoperative physical function, Hip abductor strength, Contralateral side

## Abstract

**Purpose:**

This study aimed to clarify the association between preoperative physical function and discharge destination after total hip arthroplasty (THA).

**Methods:**

This retrospective study included patients who underwent primary unilateral THA for hip osteoarthritis. Preoperative physical function was assessed via maximal isometric muscle strength (hip abduction and knee extension) and comfortable walking speed. The patients were divided into two groups according to the discharge destination (home or transfer to other facilities). Multivariate logistic regression analysis was used to identify preoperative physical function associated with discharge destination.

**Results:**

Of the 174 patients, 120 were discharged directly to home, and 54 were transferred to other facilities. Those transferred to other facilities were significantly older, more likely to live alone, and had a longer operation time. In addition, they demonstrated lower hip abductor strength on both sides and lower knee extensor strength on the operative side. Multivariate logistic regression analysis revealed that hip abductor strength on both sides, not knee extensor strength, was independently associated with the discharge destination. The largest area under the receiver operating characteristic curve was 0.668 for the hip abductor strength of the contralateral side. The optimal cutoff point was revealed to be 0.035 kgf·m/kg and 0.031 kgf·m/kg for the operative and contralateral sides, respectively.

**Conclusions:**

Preoperative hip abductor strength, particularly on the contralateral side with a cutoff value of 0.031 kgf·m/kg, could be a predictor of discharge destination after unilateral THA. Our findings would be useful in planning rehabilitation programs.

## Introduction

Total hip arthroplasty (THA) is an effective treatment for reducing hip pain and restoring hip function in individuals with hip osteoarthritis (OA). Maximizing functional mobility is one of the important goals of rehabilitation in the acute phase after THA [1]. While some patients show early functional recovery and are discharged directly home, others often require prolonged length of hospital stay and/or transfer to rehabilitation facilities due to insufficient functional recovery. Early prediction of poor functional recovery is useful in planning postoperative treatment and reducing the length of hospital stay.

Preoperative physical status is a well-recognized and important determinant of postoperative rehabilitation courses and functional recovery. In patients undergoing THA, preoperative physical functions, such as lower limb muscle strength and walking speed, have been reported to be predictive factors of postoperative outcomes, including walking ability and time to walking independence [2–7]. Hence, the discharge destination after THA may also be affected by preoperative physical function. To date, some studies have investigated factors associated with discharge destination after THA and identified some factors such as age, sex, and social support [8–13]; however, the association between preoperative physical function and discharge destination after THA remains unclear. If preoperative physical function is associated with the discharge destination, it would be useful for preoperative prediction of risk stratification for poor functional recovery and postoperative planning. This study aimed to answer: (1) whether each of the preoperative physical functions, lower limb muscle strength on the operative and contralateral sides and walking speed, would be a predictor of discharge destination; (2) what is the cutoff value for the preoperative physical function that predicts discharge destination.

## Material and methods

### Study population

**Fig. 1:**
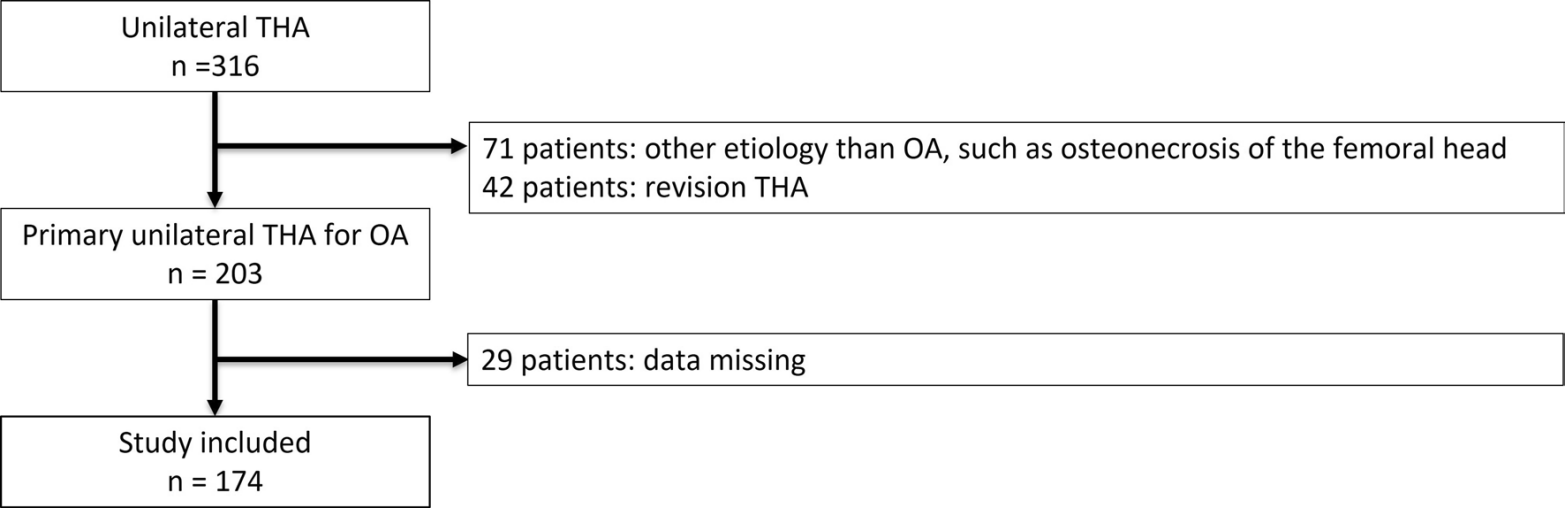
Flowchart of the patient selection procedure. OA, osteoarthritis; THA, total hip arthroplasty

We screened 316 consecutive patients who underwent unilateral THA at a University Hospital between June 2016 and June 2020, and enrolled 203 who underwent primary unilateral THA for hip OA. Of the 203 patients, 29 who did not complete the preoperative physical function assessment (lower limb muscle strength and comfortable walking speed) were excluded. Thus, 174 patients were included in the final analysis (Fig. 1). THA was performed via a standard posterior approach in all patients. The postoperative rehabilitation program started on the day after the surgery and included a range of motion exercises, muscle strengthening exercises, walking training, and other functional activities. The general goal of in-hospital rehabilitation was to be able to walk with a cane and to walk up and down stairs. This study was approved by the Institutional Review Board of our institution (approval number 2020-0232).

### Variables and outcomes

Clinical details of the presentation and demographic data were obtained from the electronic medical records. The hip function was evaluated using the Japanese Orthopaedic Association (JOA) hip score. The JOA hip score consists of four subcategories: pain, range of motion, walking ability, and activities of daily living, with a maximum score of 100 points [14]. The contralateral side hip status was classified into three groups based on the radiographic change assessed by Kellgren–Lawrence (KL) grade: normal with KL grade ≤ 2, hip OA with KL grade > 2, and already treated with THA [15, 16].

### Preoperative physical function assessment

Preoperative physical function was assessed via lower limb muscle strength and comfortable walking speed. Hip abductor and knee extensor strength on the operative and contralateral sides were measured using a handheld dynamometer with a restraining belt (μTas; ANIMA, Tokyo, Japan), as described previously [3]. For the hip abductor, patients laid in a supine position with neutral hip adduction/abduction, and the force sensor was placed 5 cm proximal to the lateral epicondyle of the femur [3]. For the knee extensor, patients sat on the edge of a platform with the knee at 60° flexion, and the force sensor was placed at the anterior part of the lower leg, 5 cm proximal to the lateral malleolus. The maximum voluntary isometric contraction for 5 s was performed twice, and the greatest value was used for the analyses. The torque (kgf·m) was calculated by multiplying the dynamometer value (kgf) and lever arm and was expressed as a percentage of the body weight (kgf·m/kg). Comfortable walking speed was measured over the middle 10 m of a 16-m walkway. The measurements were taken twice, and the highest value was used in the analyses.

### Data analyses

Since all variables showed a non-normal distribution, nonparametric tests were used. Continuous variables were expressed as medians [interquartile ranges], and categorical variables are expressed as numbers (%).

Patients were divided into two groups according to the discharge destination: home or non-home groups. Between-group differences were evaluated using the Mann–Whitney U test and Chi-squared test for continuous and categorical variables, respectively. Multiple logistic regression analysis was also performed to investigate whether preoperative physical function was associated with discharge destination. In the multiple logistic regression analysis, age, sex, living alone, JOA hip score, contralateral hip status, and operation time were included as adjustment variables based on clinical importance and previous reports. For the factors that were significant in the multivariate analysis, the optimal cutoff value was defined as the point on the receiver operating characteristic (ROC) curve where Youden’s index (sensitivity + specificity − 1) was the highest [17].

All statistical analyses were performed via SPSS version 29.0 (IBM Japan, Inc., Tokyo, Japan). Statistical significance was set at *p* < 0.05.

## Results

Table 1 shows the patient characteristics. Of the 174 patients, 120 (69.0%) were discharged directly home, and 54 (31.0%) were transferred to other facilities to continue undergoing in-hospital rehabilitation. Table 2 shows the results of the univariate analysis between the two groups. Patients in the non-home group were older, more likely to live alone, and had longer operation time. Similarly, they demonstrated lower hip abductor strength on both sides and lower knee extensor strength on the operative side. Knee extensor strength on the contralateral side and comfortable walking speed did not differ between the two groups. Moreover, multivariate analysis revealed that hip abductor strength on both sides was a significant independent factor associated with discharge destination (Table 3). The optimal cutoff value was 0.035 kgf·m/kg and 0.031 kgf·m/kg for the operative and contralateral sides, respectively (Table 4).

**Table 1:** Patient characteristics

	Overall
Age, years	65.0 [57.8–73.0]
Sex, women	142 (81.6)
BMI, kg/m^2^	23.5 [21.4–26.4]
Living alone	35 (20.1)
JOA hip score, points	66.0 [54.0–79.0]
*Contralateral hip status*	
Normal hip	77 (44.3)
Hip OA	59 (33.9)
Treated with THA	38 (21.8)
*Preoperative physical function*	
Hip abductor strength on operative side, kgf·m/kg	0.037 [0.024–0.058]
Hip abductor strength on contralateral side, kgf·m/kg	0.043 [0.031–0.055]
Knee extensor strength on operative side, kgf·m/kg	0.071 [0.049–0.097]
Knee extensor strength on contralateral side, kgf·m/kg	0.083 [0.063–0.108]
Comfortable walking speed, m/s	0.84 [0.40–1.09]
Operation time, min	110.0 [89.8–140.0]
Blood loss during surgery, g	447.0 [298.3–657.3]
Postoperative length of stay, days	17.0 [15.0–21.0]
Discharge home	120 (69.0)

BMI, body mass index; JOA, Japanese Orthopaedic Association; OA, osteoarthritis; THA, total hip arthroplasty

**Table 2:** Between-group comparison

	Home group	Non-home group	*p* value
Age, years	64.0 [56.5–71.0]	67.0 [59.0–77.0]	0.031
Sex, women	102 (85.0)	40 (74.1)	0.085
BMI, kg/m^2^	23.5 [21.4–25.6]	23.7 [21.5–27.1]	0.425
Living alone	16 (13.3)	19 (35.2)	< 0.001
JOA hip score, points	65.0 [53.5–79.0]	70.0 [54.0–82.0]	0.576
*Contralateral hip status*			0.367
normal hip	57 (47.5)	20 (37.0)	
hip OA	37 (30.8)	22 (40.7)	
treated with THA	26 (21.7)	12 (22.2)	
*Preoperative physical function*			
Hip abductor strength on operative side, kgf·m/kg	0.041 [0.028–0.061]	0.027 [0.019–0.044]	< 0.001
Hip abductor strength on contralateral side, kgf·m/kg	0.045 [0.033–0.058]	0.033 [0.024–0.045]	< 0.001
Knee extensor strength on operative side, kgf·m/kg	0.075 [0.050–0.099]	0.059 [0.046–0.084]	0.049
Knee extensor strength on contralateral side, kgf·m/kg	0.088 [0.066–0.116]	0.076 [0.057–0.101]	0.059
Comfortable walking speed, m/s	0.90 [0.52–1.11]	0.79 [0.39–1.04]	0.335
Operation time, min	106.0 [85.8–126.0]	131.0 [99.8–154.5]	< 0.001
Blood loss during surgery, g	418.0 [279.9–614.0]	485.5 [302.3–819]	0.144
Postoperative length of stay, days	17.0 [14.5–20.0]	19.0 [15.0–22.0]	0.212

BMI, body mass index; JOA, Japanese Orthopaedic Association; OA, osteoarthritis; THA, total hip arthroplasty

**Table 3:** Multiple logistic regression analysis determining preoperative physical function associated with discharge destination

	OR	95% CI	*p* value
Hip abductor strength on operative side, per 0.01 kgf·m/kg decrease	1.180	1.026–1.357	0.021
Hip abductor strength on contralateral side, per 0.01 kgf·m/kg decrease	1.248	1.019–1.527	0.032
Knee extensor strength on operative side, per 0.01 kgf·m/kg decrease	1.073	0.964–1.194	0.200
Knee extensor strength on contralateral side, per 0.01 kgf·m/kg decrease	1.071	0.963–1.191	0.209
Comfortable walking speed, per 1 m/s decrease	1.634	0.632–4.225	0.311

Each variable was adjusted with age, sex, living alone, JOA hip score, contralateral hip status and operation time

CI, confidence interval; OR, odds ratio

**Table 4:** Cutoff values for predicting non-home discharge obtained from receiver operating characteristic curve

	Area under curve	Cutoff point	Sensitivity (%)	Specificity (%)	Positive predictive value (%)	Negative predictive value (%)
Hip abductor on operative side, kgf·m/kg	0.666	0.035	68.5	60.8	40.4	68.5
Hip abductor on contralateral side, kgf·m/kg	0.668	0.031	81.7	48.1	54.2	48.1

## Discussion

In this study, we investigated the association between preoperative physical function and postoperative discharge destination in patients who underwent unilateral THA. Our results suggest that preoperative hip abductor strength, not knee extensor strength or comfortable walking speed, could be an independent predictor of the discharge destination after unilateral THA. Previous studies have identified factors associated with the discharge destination after THA, such as age, sex, and social support. Our findings would be useful in predicting early those with a high risk of non-home discharge and in further reducing the length of hospital stay.

Some preoperative physical functions were independently associated with the discharge destination in this study. The impact of preoperative physical function on the clinical course after THA has been reported. Our findings may be supported by previous studies that have reported associations between preoperative physical function and short-term clinical courses, such as walking independence within a few days to 2 weeks after THA [2, 6, 7, 18]. It is reasonable to assume that poor preoperative physical function is associated with a risk of late functional recovery and, consequently, difficulty in discharge to home. Our findings suggest that preoperative physical function is also important when considering discharge destination after THA.

In contrast, comfortable walking speed was not associated with discharge destination in this study. This result is inconsistent with those of some studies that reported an association between preoperative walking speed and clinical course [6, 7]. There seemed to be two potential factors for this inconsistency: differences in measurement conditions and the study populations. First, only comfortable speed was used in the study because patients were often unable to walk “fast” due to reasons such as pain and fear of falling, whereas Shibuya et al. observed a higher predictive capacity of “maximal” speed than “usual” speed [6]. Second, the overall population in this study (comfortable speed of 0.84 m/s at the median) might be poorer than those in a previous study that reported a cutoff point of 1.0 m/s as a predictor of delayed functional recovery [7]. Moreover, our results suggested that not walking speed but hip abductor strength could predict discharge destination. It has been indicated that preoperative muscle strength measurements could be useful for postoperative prediction, even in those with relatively decreased functional mobility.

The largest area under the ROC curve in the hip abductor strength on the contralateral side suggested that this indicator would be particularly useful in predicting the discharge destination. This result is in line with those of previous studies focusing on the importance of contralateral lower limb functions [4, 5, 19, 20]. One potential reason may be that the contralateral limb often plays a key role in activities of daily living early after THA since non-operative side-dominant weight loading is common in patients with hip OA before and after THA [21, 22]. Inhibition in muscle strength on the operative side due to pain may also be related to this result. Since muscle strength in the affected limb is influenced more by pain than by muscle quantity and quality [23], muscle strength on the contralateral side could be a relatively reliable indicator reflecting physical function independent of pain.

Our results showed that preoperative muscle strength was associated with discharge destination independent of the contralateral hip status, assessed in three groups: normal, OA, and THA. These findings emphasize the importance of rehabilitation, particularly muscle strengthening training, in various phases, including before and after THA. First, preoperative rehabilitation, the so-called prehabilitation, including the contralateral side, would be crucial for all the patients scheduled for THA. In addition, for those with contralateral THA, postoperative rehabilitation to maximize muscle strength on both sides will also be important for the non-operative side. Of interest is that unilateral THA has been reported to improve contralateral hip function in certain populations [16]. Although more research is needed on the interaction between limbs, it is important to consider the affected and the contralateral sides in rehabilitation before and after THA.

There are some limitations in this study. This was a retrospective, single-center study in a small population. In addition, the study population included only Asian patients after THA. Although our results provided insight into the impact of preoperative muscle strength on the discharge destination after THA, further studies are required to validate our findings.

In conclusion, preoperative hip abductor strength on the contralateral side with a cutoff value of 0.031 kgf·m/kg could be an independent predictor of discharge destination in individuals undergoing unilateral THA for hip OA. Our findings may be useful for risk stratification and planning of individual rehabilitation programs.
